# Mutations in rpoB and katG genes of multidrug resistant mycobacterium tuberculosis undetectable using genotyping diagnostic methods

**DOI:** 10.11604/pamj.2017.27.145.10883

**Published:** 2017-06-28

**Authors:** Faustinos Tatenda Takawira, Racheal Shamiso Dube Mandishora, Zephaniah Dhlamini, Ellen Munemo, Babill Stray-Pedersen

**Affiliations:** 1 Department of Applied Biology and Biochemistry, National University of Science and Technology (NUST), Bulawayo, Zimbabwe; 2 Department of Microbiology, College of Health Sciences, University of Zimbabwe, Mazowe Street, Parirenyatwa Complex, Avondale, Harare, Zimbabwe; 3 National Microbiology Reference Laboratory, Harare Central Hospital, Southerton, Harare, Zimbabwe; 4 Division of Woman, Oslo University Hospital, Rikshospitalet and Institute of Clinical Medicine, University in Oslo, Norway

**Keywords:** Mutation, rpoB, katG, GeneXpert, hains genotype MTBDRplus, mycobacterium tuberculosis, sequencing, DNA and PCR

## Abstract

**Introduction:**

Tuberculosis remains the leading causes of death worldwide with frequencies of mutations in rifampicin and isoniazid resistant Mycobacterium tuberculosis isolates varying according to geographical location. There is limited information in Zimbabwe on specific antibiotic resistance gene mutation patterns in MTB and hence, increased rate of discordant results and mortality due to inappropriate antibiotic prescriptions. The rpoB and katG genes molecular markers are used for detecting rifampicin and isoniazid resistance respectively. Some mutations within these gene sequences are associated with drug resistance as they directly alter gene function. The objectives of this research was to determine the drug resistance profiles in M. tuberculosis isolates that are phenotypically resistant but not detected by the GeneXpert and MTBDRplus kit and also to detect mutations in the rpoB and katG genes which are not detected by the Hain Genotype MTBDRplus kit and GeneXpert diagnosis.

**Methods:**

PCR was used for the amplification of the rpoB and katG genes from MTB isolates collected from human clinical samples between 2008 and 2015. The genes were sequenced and compared to the wild type MTB H37Rv rpoB (accession number L27989) and kat G genes (KP46920), respectively. Sequence analysis results were compared to genotyping results obtained from molecular assays and culture results of all isolates.

**Results:**

The most frequent mutation responsible for rifampicin resistance was (25/92) S531L that was detected by using all molecular assays. Some inconsistencies were observed between phenotypic and genotypic assay results for both katG and rpoB genes in 30 strains. For these, eight codons; G507S, T508A, L511V, del513-526, P520P, L524L, R528H, R529Q and S531F were novel mutations. In addition, the I572P/F, E562Q, P564S, and Q490Y mutations were identified as novel mutations outside the rifampicin resistance determining region. In katG gene, amino acid changes to threonine, asparagine and isoleucine exhibited high degrees of polymorphism such as V473N, D311N, and L427I. The R463L (20/92) amino acid substitution was most common but was not associated with isoniazid resistance.

**Conclusion:**

These finding indicate that molecular assay kit diagnosis that is based on the rpoB and katG genes should be improved to cater for the genetic variations associated with the geographic specificity of the target genes and be able to detect most prevalent mutations in different areas.

## Introduction

Tuberculosis remains the leading cause of death worldwide with the frequency of mutations in rifampicin and isoniazid resistance varying according to geographical location [1]. Tuberculosis (TB) is caused by Mycobacterium tuberculosis; a slow growing bacterium, which requires 4-8 weeks to isolate, identify and confirm drug susceptibility tests [2–4]. The emergence and spread of multi-drug resistant (MDR-TB) and extensively drug resistant tuberculosis (XDR-TB) poses serious challenges in controlling tuberculosis. Resistance to rifampicin is an indicator of possible multi-drug resistance as 90% of rifampicin (RIF) resistant strains are also isoniazid (INH) resistant [5–7]. MDR-TB is defined as TB that is resistant to the two first line anti-TB drugs rifampicin and isoniazid [8]. MDR-TB is a challenge to TB control due to its complex diagnostic and treatment obstacles. XDR-TB is currently defined as multi-drug resistant TB to at least the two most potent anti-TB drugs, rifampicin and isoniazid in addition to any one of the fluoroquinolones and at least one of the injectable second-line drugs; capreomycin, kanamycin and amikacin [9]. XDR-TB emerges through poor diagnosis and mismanagement of MDR-TB treatment. XDR-TB is already spreading throughout all regions of the world with 9.4 million new cases and 1.7 million deaths [10–14]. Rifampicins normally inhibit transcription in bacteria by targeting RNA polymerase. The majority of rifampicin resistant strains show mutations in a portion of the RNA polymerase B subunit gene (rpoB) which specify codons 507-533.The isoniazid (INH) resistant strains have inhA promoter mutations [15–17]. In December 2010 and 2013 the World health organization endorsed the use of the commercially available Hains Genotype MTBDRplus assay kit and the GeneXpert machine for the rapid diagnosis of INH and RIF resistance. Some isolates have however been found to be resistant based on the culture methods but the genotyping machine has not been able to detect these resistant isolates [18].

**Figure 1:**
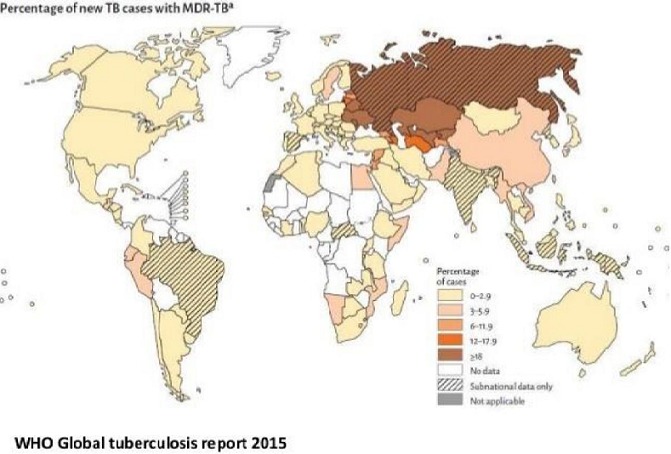
Percentage of new TB cases with MDR-TB

Although it has been observed that resistance conferring mutations are extensively studied and compiled into databases, not all mutations have been documented as observed from isolates that are phenotypically resistant but genotypically sensitive [18, 19]. These isolates have been termed as discordant and are good candidates for this study and identification of novel resistance patterns in the rpoB and katG genes [19]. There is still a need to study the mutation patterns considering that much less than half of the 46 African countries in the World Health Organization (WHO) have provided representative drug resistance data and only 10 having reported the data since 2007 [11]. This lack of genotyping data prevents the identification of unexpected and unsuspected transmission pattern including transnational spread [19]. MDR-TB cases pose a huge challenge in controlling the infection, thus indicating the need for timely and accurate laboratory diagnosis for optimizing treatment and preventing its spread [20]. The patterns and frequency of mutations in the rifampicin resistance determining region of the rpoB gene in MTB clinical isolates vary significantly according to the geographical location and (Figure 1) in addition, the available data regarding the pattern of rpoB gene mutations in MDR-TB patients in Zimbabwe limited [21]. Figure 1 [10–14]. The rpoB and katG genes in M. tuberculosis isolates that are phenotypically resistant or susceptible but not detected by the GeneXpert and MTBDR plus kit needs a better understanding of the genotypic make up and the existing mutations that are common to a particular population together with those affected assists clinicians in the choice and optimization of the most appropriate diagnostic method thus reducing the detection time [22]. In figure 1 it shows the percentage of new TB cases with MDR-TB. It is also of concern that the more widespread use of existing molecular diagnostics that miss these mutations might impose an artificial selection process where the mutants with common mutations are detected and eradicated through appropriate therapy but those with novel variants go undetected and continue to spread [22].

The study sought to detect mutations on rpoB, and katG genes of MTB from clinical samples from Zimbabwe using sequencing and subsequent analysis. Alternative diagnostic tests such as sequence based are necessary to bolster the amount of tuberculosis cases analysed for drug resistance [19]. This would require an established set of known mutations that are linked to resistance since not all mutations have been recorded as shown by isolates that lack mutations but still display resistance to antibiotics [16, 19, 23]. For example, the Hain's Genotype MTBDRplus kit has defined mutations and therefore, gene sequencing would be recommended to rule out any possible novel mutations [20]. Although certain mutations have been highly associated with drug resistance other mutations such as those recently discovered and still to be discovered could be utilized in sequence based testing to lower the discordance frequency and thus, increase the sensitivity [18]. With this in mind, significant geographical differences in the type of mutations as well as discordances have been found. This research was therefore also intended at generating more information that could be useful in enabling sequence based tests to be possibly targeted for each specific geographic region rather than using a single general test used worldwide [19, 20]. The objectives of this research was to determine the drug resistance profiles in M. tuberculosis isolates that are phenotypically resistant but not detected by the GeneXpert and MTBDRplus kit and also to detect mutations in the rpoB and katG genes which are not detected by the Hain Genotype MTBDRplus kit and GeneXpert diagnosis.

## Methods

**Ethical clearance:** This study was approved by the institutional Review Board of the Biomedical Research and Training Institute of Zimbabwe BRTI-IR.

**Sample sources and processing:** The present study was conducted in the TB laboratory at BRTI and NUST. One hundred sputum specimens were included for investigation. Hundred isolates were collected from adult patients who were at least 16 years old. These patients reported to several polyclinics in Harare from January 2008 -2015. All the samples that were archived were screened for Mycobacterium tuberculosis using the gold standard solid Lowenstein Jensen (LJ) agar.

**Culture and isolation on Lowenstein Jensen:** The Mycobacterium tuberculosis isolates were frozen in tryptic Soy Broth with 10% glycerol (TSB/glycerol) and frozen sputum were retrieved from the -70 freezer in the BRTI level 3 biosafety laboratory. After thawing, the colony suspension was homogenized by gentle mixing, then cultured on solid LJ agar. All specimens were incubated at 37^o^C and checked weekly for up to 8 weeks. When the media surface was covered with white colonies of Mycobacterium tuberculosis, they were scraped off and resuspended in a cryotube with 500ul of distilled water. The suspension was then heat killed for one hour in a water bath at 80oC. The heat killed samples could now be used outside a level 3 biosafety laboratory. The isolates were stored at -20^o^C.

**DNA extraction:** Stored DNA was used, which had been extracted using the Hains Genolyse kit where 500ul of processed sediment was used to perform the Genotype MTBDRplus version 2.0 assay according to manufacturer's instructions. The extracted DNA containing some of the discordant isolates were transported to the NUST molecular laboratory at a temperature maintained at -4^o^C. On arrival 10ul of DNA from all isolates were quantified, using the Quibit version 3.0 fluorometer assay according to manufacturer's instruction and the DNA was stored at -4^o^C.

**PCR and sequencing:** The PCR amplification of the rpoB and katG gene was performed using primers katG-F (5'-AGC TCG TAT GGC ACC GGA AC-3'), katG-R (5'-TTG ACC TCC CAC CCG ACT TG-3') and RPOB TR1 (5'-TACGGTCGGCGAGCTGATCC-3'), RPOB3R (5'-GTACGGCGTTTCGATGAACCCGAA-3') mentioned (Table 1) using Gene Amp PCR System 9700 thermal cycler. The thermocycling conditions for katG gene were one initial denaturation at 94oCfor 3 minutes followed by 30 temperature cycles involving denaturation at 94^o^C for 45sec, annealing at 65^o^C for 30sec and extension at 72^o^C for 30sec and final extension at 72^o^C for 5minutes while for the rpoB gene had 35 temperature cycles involving denaturation at 94^o^C for 1minute, annealing at 64^o^C for 1 minute and extension at 72^o^C for 5minutes. The total volume of PCR was 50ul that contained 10ul of 5ng of genomic DNA, 1ul of 20pmol of each primer, 1 ul of 200uM of dNTPs, 10 ul of 5X one Taq standard buffer (20mM Tris-HCl(pH8.9@25C), 18mM MgCl2, 22mM NH4Cl, 22mM KCl, 0.06% IGEPALCA-630, 0.05% Tween 20), 0.25ul of 1.25U of One Taq DNA polymerase (New England Biolabs), 26.75ul of molecular grade water. Genomic DNA of Mycobacterium tuberculosis H37Rv was used as a positive control, The amplified products of rpoB (412bp) and katG gene (620bp) were separated on 1% agarose gel and visualised using a UV transluminator. The PCR products were purified using the wizard SV gel and PCR clean up system and sequenced using the BioDye terminator v3.1 cycle sequencing kit (2ABI 3500XL, Applied Biosystems).

**Table 1:** Identification of the rpoB mutation for rifampicin resistant M. tuberculosis strains using GeneXpert, Hains Genotype MTBDRplus and Sequencing (Bold: novel mutations in the RRDR)

Altered codons	Nucleotide change(s)	Amino acid change(s)	No. and rate of strain with mutations
507	GGC-AGC	G507S	1
508	ACC-GCC,CCC,AAC	T508A, T508N	1
511	CTG-CCG, GTG	L511P, L511V	3
512	AGC-AAC	S512N	2
513	CAA-deletion	Q513del	1
514	TTC-deletion	F514del	2
515	ATG-deletion	M515del	3
516	GAC-deletion, GGC, TAC	D516del, D516G, D516Y	8
518	AAC-deletion, CAC	N518del, N518H	3
519	AAC-deletion	N519del	1
520	CCG-CCC, ACG	P520P, P520T	2
524	TTG-TTA	L524L	1
526	CAC- deletion, GAC, CTC	H526del, H526D, H526L	4
528	CGC-CAC	R528H	2
529	**CGA**-CAA	R529Q	1
531	**TCG**-TTG, TTC, TGG	S531L, S531W, S531F	25
533	**CTG**-CCG	L533P	1
**Mutations outside the rifampicin resistant determing region (81bp)**			
500	GCC-GAC, ACC	A500D, A500T	1
502	ATC-ACC	I502T	2
503	AAG-AAC,AAA	K503N, K503K	10
504	GAG-AAG	E504K	28
505	TTC-TTG, TTT	F505L. F505F	2
506	TTC-TTG	F506L	1
490	CAG-GAA,TAG,CAA,TAT	Q490E, Q490Y, Q490Q, Q490STOP	17
562	GAA-GAT,GCA,CAA,GGT	E562D, E562A, E562Q, E562G	9
564	CCT-CCA, delT,TCG	P564P. P564S, P564del	3
553	TCG-deletion, TCA	S553del, S553S	15
572	ATC-CCG, TTC	I572P, I572F	3
471	ATG-GTG,CTG,delAG,A-G, CAG, GAG	M471V, M471L, M471del, M471E	35
559	TGC-delT, ATT, GTC, TCC	C559del, C559C, C559Y	26
557	CGG-CAG, CAA. GGG	R557Q, R557G	7
555	TAC-CAC, TCC, TTC, TAA, TCA, ACA	Y555H, Y555S, Y555F, Y555T, Y555S, Y555stop	34
543	GCC-ACG, TAC, AC-.-CC	A543T, A543Y, A543del	12
**No mutation**			6
547	GTC-GTT,GTA, -GT, -TT	V547V, V547del	18
567	CCC-ACC, -CC	P567T, P567del	10
570	GGT-GAT	G570D	10

**Sequence analysis:** All nucleotide sequence chromatogram of clinical isolates were edited using Chromas lite, NCBI pairwise BLAST (http://www.ncbi.nlm.nih.gov/BLAST) was used for gene identification and homology searches in order to establish the microorganism and sequence alignment.

**Collation of all data:** The results which emanated from the screening of mutations/sequencing in MDR-TB were compared with results from NRML database for BACTEC MGIT MDR-TB, Hain Genotype MTBDRplus assay kit, GeneXpert and the drug sensitivity tests carried out for MDR-TB. Statistical analysis of data was performed.

## Results

**Figure 2:**
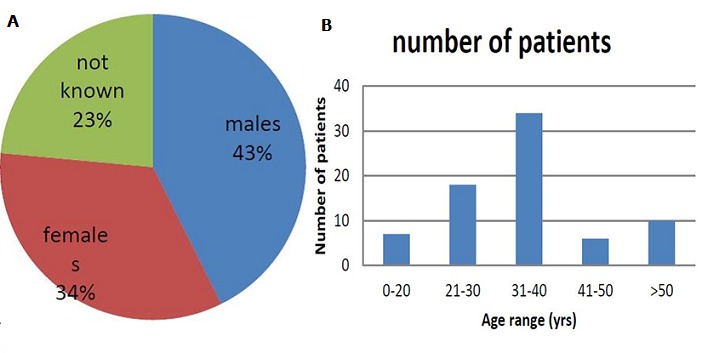
Gender and Age group distribution: [A] represents sex of the patients with a slightly higher percentage of males presenting with MDR-TB than females while [B] represents the age of screened patients which ranged from 12- 65 years old with highest number

**Figure 3:**
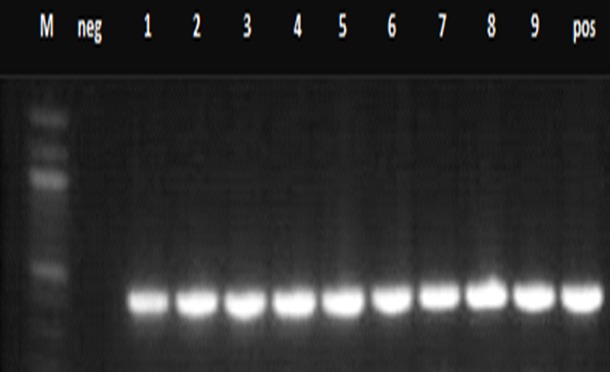
PCR amplification of rpoB gene from phenotypic MDR-TB isolates: PCR products were resolved on 1% agarose gel. M (Marker) in the figure represents the 100bp molecular weight DNA ladder. Lane 1-9 show amplification of a 412bp fragment of rpoB gene, Neg- negative control, Pos- positive control

**Figure 4:**
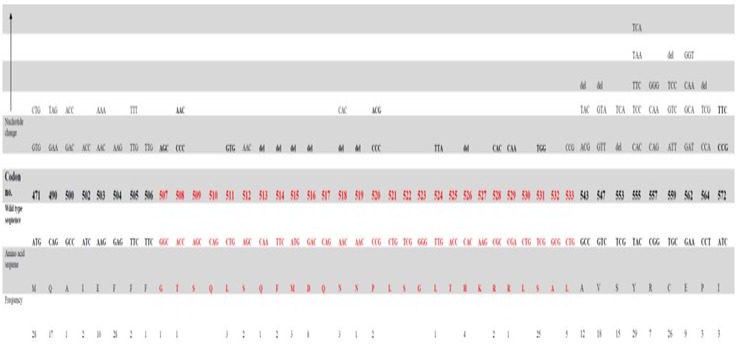
Mutations and alleles in rifampin resistant M. tuberculosis isolates reported in this study

**Figure 5:**
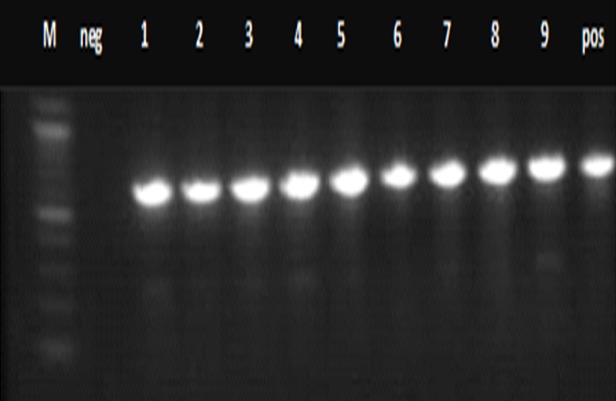
PCR amplification of katG gene from phenotypic MDR-TB isolates: PCR products were resolved on 1% agarose gel. M (Marker) in the figure represents the 100bp molecular weight DNA ladder. Lane 1-9 show amplification of a 620bp fragment of katG gene, Neg- negative control, Pos- positive control

Specimens from patients: The initial number of screened samples were one hundred, among the hundred samples, only 8 samples had negative results and those that were analysed by PCR and sequencing were 92 samples. Seventy-seven samples came from the previous study and had 15 discordant results while 15 more samples with discordant results came from the National Microbiology Reference Laboratory. All samples were sequenced and analysed including the 30 discordant isolates. There was a slight increase in the MDR-TB isolates that were detected by the LJ method as compared to those that were detected by genotypic assay (Figure 2, Figure 3). The Figure 4 shows a diverse profile of the rpoB gene mutation that was yielded from this study. Eighty-six out of 92 (93%) rifampicin resistant isolates showed mutations either within or outside the RRDR region. The majority of isolates showed mutations at codons 471, 490 (28, 31), 500, 502, 503, 504, 505, 506, 543, 547, 553 [31], 555, 557, 559, 562, 564 [4] and 572 [31] outside the RRDR with some being reported for the first time and others having been reported before. Six isolates had no mutation in the rpoB gene but had mutations in the katG gene. Two isolates had multiple deletions at codon 514, 515,516 and two isolates had point deletion at codon 518 and 526, one had another multiple deletion at codon 515, 516 and 518-519 and 5 isolates had point deletions at codon 516. Silent mutations were recorded in two isolates at codon 520 and one isolate at codon 524 (Table 1, Figure 4, Figure 5).

**Figure 6:**
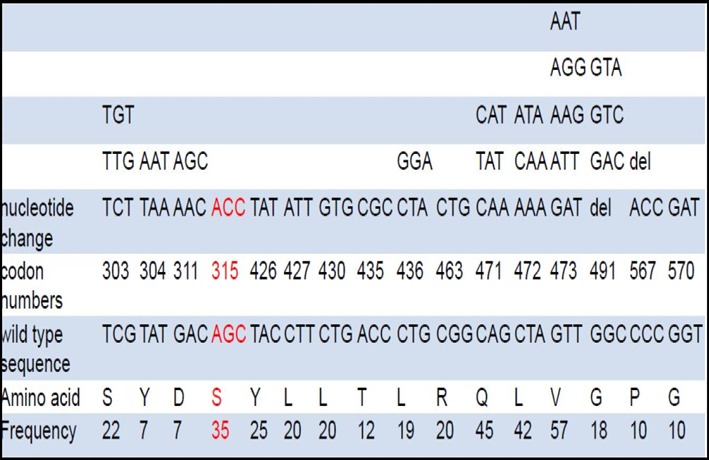
Schematic diagram showing frequent mutations in katG gene at codon 315 and other mutations outside this region are novel except for mutations at codon 463 which does not code for resistance

The Figure 6 shows that there was a diverse profile of the katG gene mutation that was yielded from this study. A higher number of isolates (57/92) 57% had mutation at codon 473 which is outside the determining region for the katG gene followed by 45 isolates at codon 471 and 472. Thirty-five isolates had mutations at codon 315 while other deletions have been observed at codon 491 on 9 isolates. Very close to the determining region for katG there were mutations at codon 303, 304 and 311 on 22 and 7 isolates giving a resistance pattern Table 2, Figure 6). Hains Genotype MTBDRplus assay was reported as MDR after resistance have been detected in both isoniazid and rifampicin, Mono Rif/ Inh when the kit detected resistance to rifampicin only or isoniazid only. For GeneXpert the result was termed Rif sensitive when the isolate had no mutation in the RRDR region while it was termed resistant when the isolate had a mutation in the RRDR region. No sample result was recorded to an isolate that had very low sputum to be used for determing resistance since low levels of sputum or DNA can result in false positive results (Table 3).

**Table 2:** Identification of the katG gene mutations for rifampicin resistant Mtuberculosis strains using Hains and Sequencing

Altered codons	Nucleotide change(s)	Amino acid change(s)	Number and rate
303	TCG-TCT, TTG, TGT	S303S, S303L, S303C	22
304	TAT-TAA, AAT	Y304stop, Y304inst	7
311	GAC-AAC, AGC	D311N, D311S	7
315	AGC-ACC	S315T	35
426	TAC-TAT	Y426Y	25
427	CTT-ATT	L427I	20
430	CTG-GTG	L430V	20
435	ACC-CGC	T435R	12
436	CTG-CTA, GGA	L436L, L436G	19
463	CGG-CTG	R463L	20
471	CAG-CAA, TAT, CAT	Q471Q, Q471Y, Q471H	45
472	CTA-AAA. CAA, ATA	L472K, L472Q, L472I	42
473	GTTGAT,ATT,AAG,AGG,ATG,AGT,TTT,TGG,TAT,GTG,GGT,TAA,AAT	V473D, V473I, V473K, V473R, V473M, V473S, V473F, V473W, V473Y, V473V, V473G, V473stop, V473N	57
491	GGC-G-C, GAC, GTC, GTA	Del491, G491D, G491V, G491V	18

**Table 3:** Comparison of culture results with genotypic methods and the codons affected Bold: Novel mutations that have been recorded and not included in the RRDR and katG gene determining region and that have been found to code for resistance, The other mutations are novel mutations

Culture	Hain MTBDRplus kit	GeneXpert Machine	PCR rpoB Sequencing Results	PCR katG Sequencing Results
MDR	Mono inh	Not determined	557, 572. 574	315, 471 472, 473
MDR	Sensitive	No sample	555, 557	463, 472, 473
MDR	Mono inh	Not determined	533, 471	315, 471, 472
MDR	Mono inh	Not determined	533	303, 315, 472, 473, 474
MDR	Sensitive	Sensitive	471. 490, 504,553, 559, 569, 570, 576	463,471, 472, 473
Sensitive	MDR	Resistant	471,531, 591, 504, 490	315,426, 427, 430, 435, 436, 453, 471, 473, 476, 491, 492
MDR	Sensitive	Sensitive	471, 504, 503	315, 471, 472, 473, 491, 493
Sensitive	Mono rif	Not determined	471, 504, 531,490	315, 463, 471, 472, 491, 494
Mono rif	Sensitive	Rif resistant	503, 470, 471, 490, 497	430, 453, 435, 436, 476, 491, 492
Sensitive	MDR	Rif sensitive	543. 547, 550, 559, 561. 567, 573	303, 315, 426, 427, 471, 472, 473, 491
MDR	Sensitive	Sensitive	No mutation	315, 426, 427, 430, 436, 473, 491
MDR	Sensitive	Sensitive	547, 553, 559, 576	
Sensitive	Mono rif	Sensitive	526,555, 559	315, 463, 471, 473, 493, 494
Sensitive	MDR	Resistant	531, 556, 562, 564, 490, 471	No result
MDR	Mono rif	Sensitive	471, 526	
Sensitive	Sensitive	Sensitive	512, 556, 559	303, 471, 472, 473
Mono rif	Sensitive	Not determined	576, 471, 504	
Mono inh	Mono inh	Rif sensitive	554, 556, 459, 472, 475	426, 429, 430, 432, 436, 443, 446, 447, 451, 453, 457, 469, 473, 476, 483, 484
Mono rif	Mono rif	Sensitive	531, 553, 555, 559, 504,490, 473, 471	315, 380, 435, 436, 437, 438, 442, 426, 429, 430,

## Discussion

Rifampicin resistance has been found to be associated with a hotspot (codon 507-533) core region called rifampicin resistant determining region (RRDR) where several authors have described the presence of common as well as novel mutations for rpoB gene especially in the hotspot region as well as outside the hotspot region but within the rpoB gene [20, 24, 25]. This diversity has been shown to demonstrate implications in the vaccines and diagnostics for TB. No information on the mutations outside the hotspot region or its diversity in the hotspot region have been recorded in Zimbabwe. In this study we reported the presence of common and novel rpoB gene mutations in clinical Mycobacterium tuberculosis isolates from Zimbabwe and their clinical and diagnostic implications in the context of a public health programme setting. All the isolates that are susceptible to rifampicin have shown wild type sequence and most of the resistant isolates showed mutations within and outside the hotspot region [26]. The emergence of multidrug resistance is a serious hurdle for clinicians, since it arises mostly due to rpoB gene mutations instead of acquiring resistant genes from other bacteria [25]. The frequency and high level of resistance to RIF have been reported across the world and has been found to vary according to geographic location [27–29]. This diversity has also been witnessed in the case of rpoB gene mutations affecting people in Zimbabwe. In figure 2 there is a slightly higher percentage of males presenting with MDR-TB than females. The age of screened patients ranged from 12- 65 with highest number of MDR-TB patients in 31-40-year age group [29, 30]. These results correspond to what other countries have witnessed and it may be attributed to lifestyle factors, delay or difficulties in accessing healthcare facilities and leave from work, Patient immunocompromised and non-compliance to treatment [18, 30].

In this study our finding was that 90% of RIF isolates were MDR-TB consistent with previous studies that say the majority of MTB isolates worldwide have mutations within the 81bp core region of the rpoB gene [20]. The most frequently mutated codon in our study was S531L (25/92), followed by D516G/Y/del (8/92) and H526D, H526L (4/92) which was similar to those reported from other clinical isolates [25, 31]. Although low frequencies of mutation in codon 516 in clinical isolates have been reported in some regions, high frequency of this mutation was detected in our study. GeneXpert and Hains Genotype MTBDRplus kit are molecular methods most used because of their high sensitivity, specificity and relatively easy to perform. This study has shown that a considerable number of isolates from clinically and bacteriologically diagnosed MDR-TB patients demonstrated uncommon mutations at codons within the hotspot (deletions from 513-519) [5, 23] and outside the hotspot as shown in table 1 and 3 that are not covered by the standard molecular diagnostic kits [32]. These patients could have missed the diagnosis had they been tested with molecular tests alone. It is of great importance that these rpoB detection kits be customised so as to make them suitable to detect locally prevalent mutations. Hence this study attempted to record the baseline information on rpoB mutations prevailing in this country. S531F (4/30) mutation of rpoB gene detected through sequencing of RIF-resistant isolate, but not by Hains Genotype MTBDRplus, is a rare mutation which has been associated with MDR-TB outbreak. This mutation lies within the hot spot region covered by probe WT 8 of the Hain Genotype MTBDRplus for rpoB codon 531 and should have been detected by the assay. The fact that it was not detected highlights the limitation of Hains Genotype MTBDRplus and GeneXpert. Amino acid changes to G507S, P520P, L524L, del526 and S531F in the hotspot region were not detected by the Hains Genotype MTBDRplus kits. Some of the deletions in and out of this region (codon 430-497 and 533-572 [23, 33, 34] have been interpreted to confer low phenotypical resistance [35] while these other mutations may have been due to polymorphism in rpoB gene of MTB [36].

The sequencing data showed silent mutation (L524L) (TTG524TTA) in an isolate that was not detetcted by GeneXpert and it was termed resistant. This isolate was found to be RIF sensitive in LJ-DST. A study from Haiti as well as many other countries have seen silent mutation across the 81bp RRDR region that were sensitive in LJ proportion method [16, 37]. From this point of view, GeneXpert probes cannot differentiate silent mutations which may cause misinterpretation of RIF susceptibility [23, 38]. Discrepancies among LJ-DST and two molecular DSTs have been associated with the detection of more resistant cases by molecular methods which may happen due to two reasons. Firstly, these two molecular methods can detect few silent mutations, which do not affect the phenotypic DST [38, 39]. Secondly, GeneXpert is a quantitative method, which predicts resistance based on ∆Ct value; so it is possible to get variable result than any qualitative method like LJ-DST [23]. There was a mutation that have been found to result in discordance results outside the hotspot region at codon 572 [31, 40, 41]. This has been recorded by other authors and has been proven to cause phenotypic resistance. Currently available molecular tests are designed in such a way as to detect known rpoB polymorphism including 531, 516, and 526 that commonly occur in MTB isolates [18]. However, this may not hold good for universal application across various settings, considering the geographic diversity of mutations. Discordant results between genotypic and phenotypic methods concerning RIF sensitivity have been recorded in this study to be 30 isolates. Some of the discrepancies that were recorded in this study such as no sample by the GeneXpert may be due to either a technical error that occurred in the strain suspension preparation before inoculation or a problem with bacterial cultures that grow poorly and the gold standard used [39]. The other mutations such as deletions in the hotspot gave frequent discrepancies between genotypic and phenotypic methods. For some authors these mutations are concordant with phenotypic resistance to RIF [5]. The other reasons resulting in discordance of molecular kits and drug susceptibility test results are that not all mechanism of resistance are known and the lack of mutation is not equal to drug susceptibility, there are also limited genes and sites targeted, Mixed population result in the emergence of resistance which may not be detected, that is in case of a hetero-resistance GeneXpert will not detect a minority resistant population when most of the population is susceptible eg S531L can only be picked up when 66% of population must be resistant, L533P when 100% of population must be resistant [30, 42]. Not all mutations are associated to phenotypic resistance especially most of the silent mutation which result in no change in protein. These discordant results have clinical significance and rifampicin resistance detected by GeneXpert must be confirmed by sequencing.

In most of RIF resistant isolates had a WT band (found in drug susceptible strains) that was missing but corresponding MUT band (found in drug resistant strains) was present. This banding pattern is likely to be a result of mutations associated with drug resistance. Another possible result pattern represents a silent mutation that does not result in amino acid change or may indicate the presence of less common mutations at the rpoB gene that cannot be detected by the current version 2 of the Genotype MTBDRplus kit [43–45]. Another banding pattern that was frequent on 10 isolates was that of no wild type band and no corresponding mut bands which is a pattern that represents the presence of an unknown mutation causing resistance but not being detected by the kit. Two isolates showed hetero-resistance where the banding pattern had all wild type bands present without mutant bands. Another rpoB gene mutation not detected by the Hains Genotype MTBDRplus was a deletion at codons 513-519. This region of the rpoB gene is situated between two probes (WT 4 and 5) and, thus, can be missed due to their overlap. Deletion at rpoB 518 is a rare mutation and has previously been reported especially in Asian countries as missed by another line probe test, the Inno-LiPA Rif.TB, (Innogenetics, Belgium) [46–48]. Another gene that is associated with resistance in MTB is the katG. The katG (CGG-CTG)(Arg-Leu) R463L (20/92) amino acid substitution is the most common polymorphism and is not associated with INH resistance [6]. The majority of deletions in the katG gene account for inactivation of the catalase peroxidase leading to isoniazid resistance e.g at codon 491 as observed in our study [43]. Amino acid changes to threonine, asparagines and isoleucine exhibit high degrees of polymorphism e.g V473N, D311N, L427I [44]. Among the phenotypic INH-resistant strains, one was found with S303L and another with S315L mutation upon katG sequencing. Though codon 303 of katG lies beyond the hot spot regon, S303L has been reported as a rare mutation in MTB strains from Myanmar. Sequencing for katG mutations in our samples confirmed that a large number of our INH- resistant strains which were not detected by MTBDRplus did not harbour mutations at codon 315, the locus targeted by MTBDRplus. Resistance in such strains may be due to mutations in regions other than codon 315 of katG (S303L in one of our isolates).

## Conclusion

Our findings showed that the occurrence of specific rpoB mutations varied considerably in RIF-resistant M. tuberculosis isolates. The data have important implications for designing region-specific rapid methods for detecting majority of RIF-resistant strains. This study showed that MTBDRplus had a high detection rate for rifampicin and isoniazid resistance. Additional probes need to be included in the assay to improve the detection of isoniazid resistant Mycobacterium tuberculosis strains. Another important about these findings are that they showed us loop holes in the performance of GeneXpert. These observations also show that relying on GeneXpert results may be disastrous step for TB control programs as it gives alarmingly high error/false negative/ false positive results and the resistant MTB isolates are false labelled as susceptible or resistant thereby making the program managers complacent and underestimating the threat of MDR-TB.

**Recommendations:** Although this is a small sample size, the high discrepancy between Rif and INH resistance indicates the need for a genotypic assay that can detect both Rif and INH resistance that are region specific. Further analysis on these discordant results should be done as a way of understanding the reason behind. A better understanding of the clinical implications of a positive molecular test in the face of negative culture vice versa is required. There is a need for the development of kits targeting other genes, region-specific kits to be developed targeting rpoB mutations (in and out of the RRDR) prevalent in the region. GeneXpert should be used for the screening of MTB but should be used together with another assay that targets other genes in cases of mono-resistance to isoniazid.

### What is known about this topic

- rpoB and katG genes are molecular markers used for detecting rifampicin and isoniazid resistance;
- Mutations in rifampicin and isoniazid vary according to geographical location and alter gene function;<:li>
- 90% of rifampicin resistance are isoniazid resistance.

### What this study adds

- This research generated more information that could be useful in enabling sequence based tests to be possibly targeted for each specific geographic region rather than using a single general test used worldwide (Improvement of the diagnostic kits) ;
- This research adds more information on mutations that are not identified by genotyping machines which are observed as isolates that are phenotypically resistant but genotypically sensitive vice versa. This data also increases chances of identifying unexpected and unsuspected transmission pattern including transnational spread;
- It adds more information to the limited data in Zimbabwe on specific antibiotic resistance gene mutations patterns. Zimbabwe will be added to the list of WHO African countries that have supplied representative drug resistance data pattern since only 10 countries have reported their data excluding Zimbabwe. The inclusion of these mutations reduces the imposition of an artificial selection process where the mutants with common mutations are detected and eradicated through appropriate therapy but those with novel variants go undetected and continue to spread.

## Competing interests

The authors declare no competing interest.
